# Respiratory syncytial virus-induced acute and chronic airway disease is independent of genetic background: An experimental murine model

**DOI:** 10.1186/1743-422X-2-46

**Published:** 2005-05-25

**Authors:** Susana Chávez-Bueno, Asunción Mejías, Ana M Gómez, Kurt D Olsen, Ana M Ríos, Mónica Fonseca-Aten, Octavio Ramilo, Hasan S Jafri

**Affiliations:** 1 Division of Pediatric Infectious Diseases, Department of Pediatrics, The University of Texas Southwestern Medical Center at Dallas and Children's Medical Center Dallas, Dallas, Texas, USA; 2 Department of Pathology, The University of Texas Southwestern Medical Center at Dallas and Children's Medical Center Dallas, Dallas, Texas, USA

**Keywords:** Viral pneumonia, mouse model, airway hyperresponsiveness, PCR, cytokines

## Abstract

**Background:**

Respiratory syncytial virus (RSV) is the leading respiratory viral pathogen in young children worldwide. RSV disease is associated with acute airway obstruction (AO), long-term airway hyperresponsiveness (AHR), and chronic lung inflammation. Using two different mouse strains, this study was designed to determine whether RSV disease patterns are host-dependent. C57BL/6 and BALB/c mice were inoculated with RSV and followed for 77 days. RSV loads were measured by plaque assay and polymerase chain reaction (PCR) in bronchoalveolar lavage (BAL) and whole lung samples; cytokines were measured in BAL samples. Lung inflammation was evaluated with a histopathologic score (HPS), and AO and AHR were determined by plethysmography.

**Results:**

Viral load dynamics, histopathologic score (HPS), cytokine concentrations, AO and long-term AHR were similar in both strains of RSV-infected mice, although RSV-infected C57BL/6 mice developed significantly greater AO compared with RSV-infected BALB/c mice on day 5. PCR detected RSV RNA in BAL samples of RSV infected mice until day 42, and in whole lung samples through day 77. BAL concentrations of cytokines TNF-α, IFN-γ, and chemokines MIG, RANTES and MIP-1α were significantly elevated in both strains of RSV-infected mice compared with their respective controls. Viral load measured by PCR significantly correlated with disease severity on days 14 and 21.

**Conclusion:**

RSV-induced acute and chronic airway disease is independent of genetic background.

## Background

Human respiratory syncytial virus (RSV) is classified in the genus *Pneumovirus*, subfamily *Pneumovirinae*, family *Paramixoviridae*; and is a major cause of lower respiratory tract infection (LRTI) in young children and the elderly [1]. RSV LRTI is associated with increased risk of long-term recurrent wheezing [2-5], however, the pathogenesis of this relationship is not well understood. RSV LRTI elicits a host response including the release of inflammatory mediators and recruitment of different cell populations. The genetic variability of the host response might partially explain the different susceptibilities of individual patients to the acute and long-term effects of RSV infection, as suggested by the higher rates of RSV hospitalization among Native American and Alaskan Native children compared with other groups [6].

Animal models facilitate the study of RSV-induced acute and long-term disease in a more controlled manner. Our laboratory has previously established a mouse model of RSV-induced acute and long-term airway disease [7]. The present studies were designed to characterize the influence of mouse genetic background and the dynamics of viral replication on the chronic manifestations of RSV infection. The BALB/c mouse strain is one of the most commonly used for RSV experimental models, however, C57BL/6 mice frequently provide background for transgenic strains of mice. Therefore characterizing and establishing a comprehensive model of acute and long-term RSV disease in C57BL/6 is essential to further understanding the pathogenesis of RSV disease.

## Results

### 1. RSV alone induces airway obstruction (AO) and airway hyperresponsiveness (AHR) in both C57BL/6 and BALB/c mice

**Figure 1:**
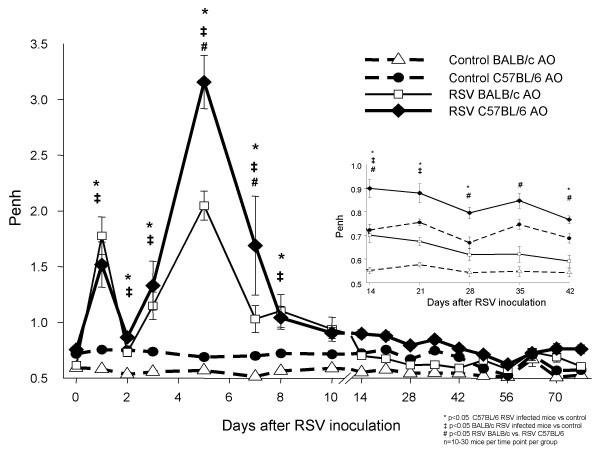
**Effect of RSV on airway obstruction (AO) in two mouse strains**. BALB/c (△) and C57BL/6 (●) mice were inoculated with sterile 10% EMEM (control) and were compared with RSV A2 infected BALB/c (□) and C57BL/6 (◆) mice to evaluate differences in airway obstruction (AO), by measuring Penh via whole-body plethysmography. Penh values are presented as means ± SEM. Comparisons were made by t-test when data normally distributed, or by Mann-Whitney Rank sum test when data were not normally distributed.

**Figure 2:**
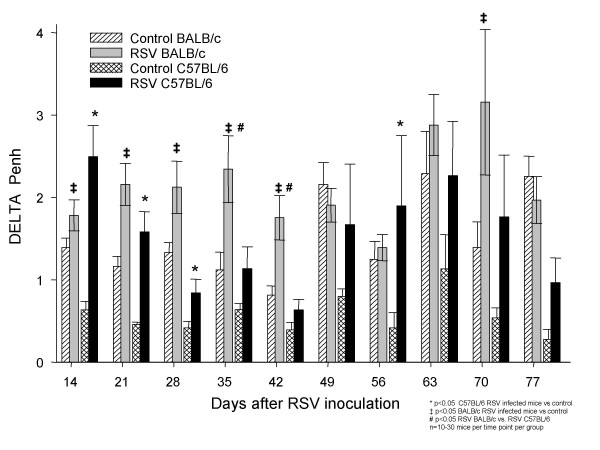
**Airway Hyperresponsiveness (AHR) in BALB/c and C57BL/6 Mice**. Data presented as Delta Penh values which is the difference between pre-and post methacholine Penh for each group of mice, in sham inoculated () and RSV inoculated () BALB/c mice, and sham inoculated () and RSV inoculated () C57BL/6 mice from days 14 to 77. Values represent the mean SEM from 10–30 mice per group. Data shown are the result of four separate experiments. p < 0.05, comparison by t-test when data normally distributed, or by Mann-Whitney Rank sum test when data were not normally distributed.

RSV infection alone, without allergic pre-sensitization induced AO in both strains of mice as demonstrated by significantly increased enhanced pause (Penh) values compared with uninfected controls. Baseline Penh values increased transiently on day 1 after RSV inoculation in both strains, decreased by day 2, but continued to be significantly greater than in controls. Airway obstruction increased again and peaked on day 5, when C57BL/6 RSV-infected mice showed significantly higher Penh values than RSV-infected BALB/c mice (p < 0.001) (Figure 1). AO decreased thereafter during the first two weeks after RSV inoculation but remained significantly greater than the respective controls in both strains, for 21 days in BALB/c and 28 days in C57BL/6 mice (Figure 1 inset). RSV infection also induced AHR in both strains as evidenced by a greater difference between pre- and post-methacholine Penh values (delta Penh) compared with controls. Significantly increased AHR was persistently present for 42 days post-inoculation in BALB/c mice, while C57BL/6 mice showed significantly increased AHR for up to 28 days post-inoculation (Figure 2).

### 2. C57BL/6 and BALB/c mice demonstrate acute and persistent inflammatory changes after RSV infection

**Figure 3:**
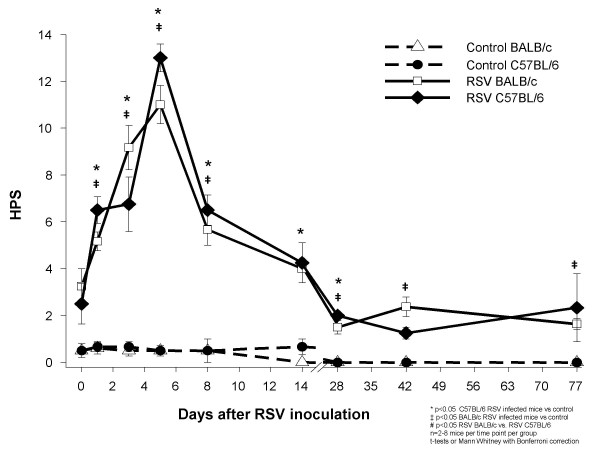
**Comparison of acute and long-term histopathologic scores after RSV inoculation**. BALB/c (△) and C57BL/6 (●) mice were inoculated with sterile 10% EMEM (control) and were compared with RSV A2 infected BALB/c (□) and C57BL/6 (◆) mice. Serial formalin fixed lung samples were obtained between day 0 (+2 hours) and day 77 after inoculation. HPS scores are represented as means ± SEM. p < .05, by t-test when data normally distributed, or by Mann-Whitney Rank sum test when data were not normally distributed.

**Figure 4:**
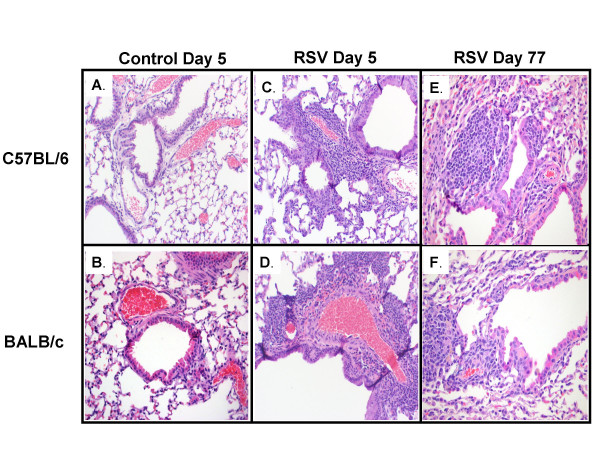
**RSV induced histopathology**. Lung specimens were harvested on days 5 and 77 from groups of C57BL/6 and BALB/c mice inoculated with sterile medium (control) or RSV. Sections from control C57BL/6 and BALB/c mice above (4A and 4B, respectively), show rare, scattered, small lymphocytic infiltrates on day 5 after inoculation with medium, similar to sections of control mice harvested 77 days after (not shown). Acute and chronic inflammatory infiltrates, surrounding airways and vessels are demonstrated in RSV-inoculated mice of both strains, on days 5 and 77 after inoculation (4C to F).

RSV-inoculated C57BL/6 and BALB/c mice, compared with their controls, showed greater histopathologic scores (HPS) which peaked on day 5 after RSV inoculation (Figures 3, 4A–D). Although the acute inflammatory changes observed in both strains gradually declined, RSV-infected mice had significantly greater HPS than the sham-inoculated controls for up to 77 days post-inoculation (Figures 3, 4E and 4F).

### 3. RSV infection induces similar cytokine production in the respiratory tract of C57BL/6 and BALB/c mice

**Figure 5:**
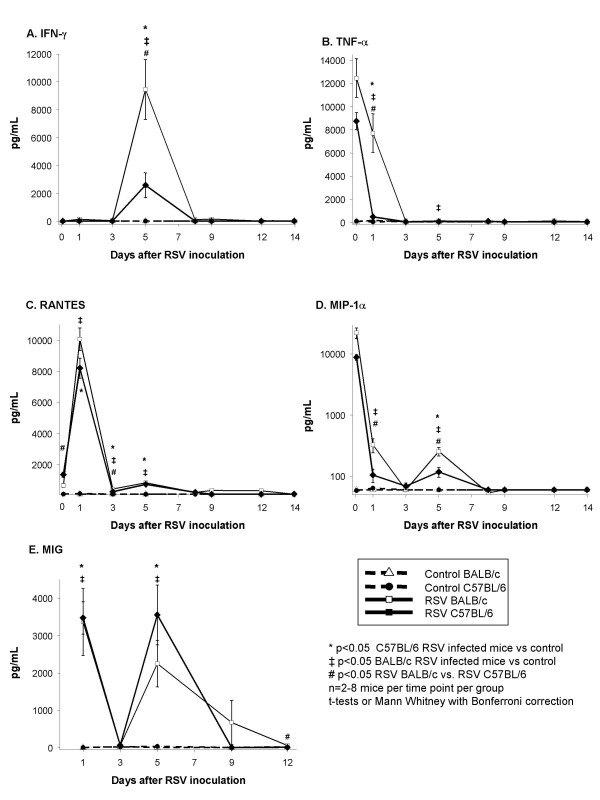
**Cytokine and chemokine concentrations in bronchoalveolar lavage (BAL) samples after RSV inoculation**. BAL samples were obtained from BALB/c (△) and C57BL/6 (●) mice inoculated with sterile 10% EMEM (control), and RSV A2 infected BALB/c (□) and C57BL/6 (◆) mice, to measure concentrations of pro-inflammatory cytokines (A) IFN-γ and (B)TNF-α; and the chemokines (C) RANTES, (D) MIP-1α, and (E) MIG. Values presented in means ± SEM pg/ml. p < .05, by t-test when data were normally distributed, or by Mann-Whitney Rank sum test when data were not normally distributed.

BAL concentrations of TNF-α, IFN-γ, MIG, RANTES, and MIP-1α followed similar dynamics in both strains of mice during the acute phase of the infection (Figure 5A–E). Overall, there was a trend for greater BAL cytokine concentrations of IFN-γ, TNF-α, RANTES and MIP-1α in RSV-infected BALB/c mice compared with C57BL/6 mice. (Figure 5A–E). No significant differences were observed in BAL concentrations of IL-4 and IL-10 between controls and infected mice of both strains at any time point evaluated (data not shown).

### 4. RSV load dynamics

#### 4a. RSV loads measured by plaque assay in BAL samples follow similar dynamics in both C57BL/6 and BALB/c mice

**Figure 6:**
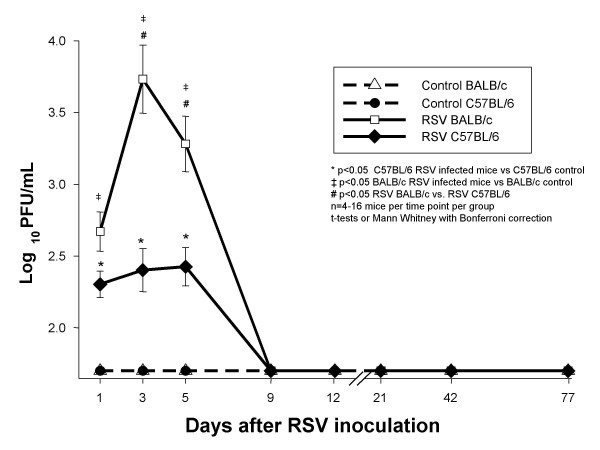
**RSV loads in BAL samples measured by the plaque assay method**. Groups of 4–16 BALB/c (△) and C57BL/6 (●) mice per group per time point were inoculated intranasally with sterile 10% EMEM (control), and were compared with RSV A2 infected BALB/c (□) and C57BL/6 (◆) mice. Viral load was determined by HEp-2 plaque assay in BAL samples. Data are presented as mean ± SEM Log10 PFU/ml of BAL. p < .05, by t-test when data normally distributed, or by Mann-Whitney Rank sum test when data were not normally distributed.

**Figure 7:**
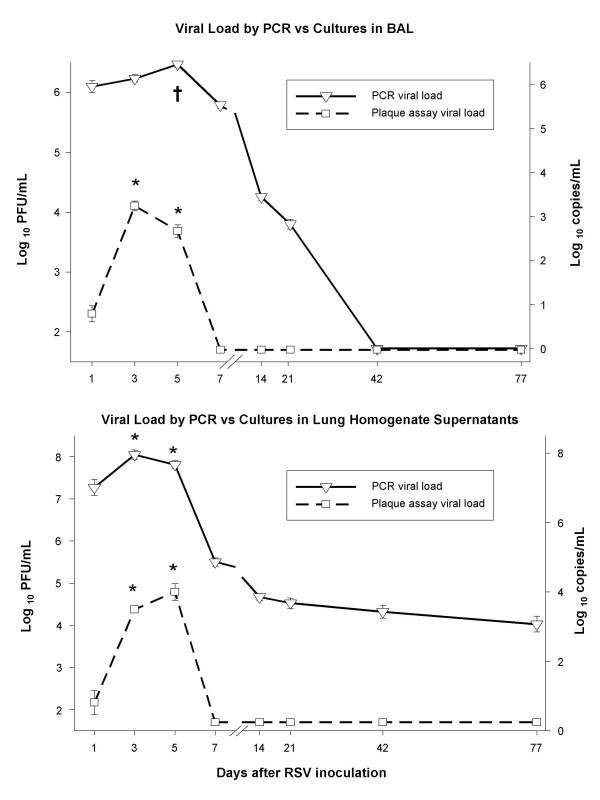
**RSV loads in RSV infected BALB/c mice BAL and lung supernatant samples measured by PCR vs. plaque assay**. RSV loads measured by PCR in BAL samples (▽) remain positive up to 42 days after inoculation, while viral loads measured by plaque assay (□) become negative on day 7 post-inoculation (upper panel). Viral load measured in lung supernatants by the plaque assay also become undetectable by day 7 after inoculation, whereas RSV loads measured by PCR in lung supernatants remain detectable throughout all the time points evaluated (lower panel). All pair-wise multiple comparisons made by One-Way ANOVA. † p < 0.01 between D1 and D5 and *p < 0.05 comparing D1 with D3 and D5.

On day 1 after RSV inoculation, RSV loads in BAL samples from both C57BL/6 and BALB/c mice were significantly greater than in controls by plaque assay (Figure 6). Compared with day 1, plaque assay RSV loads peaked on days 3–5 after inoculation in both strains representing active viral replication (p = 0.002 for day 1 vs days 3–5 in BALB/c mice; ANOVA), and were significantly greater in BALB/c than in C57BL/6 mice (Figure 7). BAL RSV loads declined below the limit of detection by day 7 and remained undetectable through day 77 post-inoculation.

#### 4b. Real Time PCR (RLT-PCR) demonstrates RSV RNA after the virus is no longer detectable by plaque assay

To further characterize the dynamics of RSV infection, we used RLT-PCR in parallel with plaque assays, to measure RSV loads in both BAL samples and lung homogenate supernatants. These experiments were initially conducted in BALB/c mice. RSV loads measured by RLT-PCR and plaque assay in both BAL and lung supernatant samples peaked on days 3 to 5 after inoculation. Similar to plaque assay, RSV load by RLT-PCR also demonstrated a significant increase in viral copies between day 1 and days 3–5, likely demonstrating active replication (Figure 7). In contrast to RSV loads measured by plaque assay, which became undetectable by day 7 after inoculation (Figure 7, dashed-line plots), RSV loads measured by RLT-PCR remained positive for 42 days in BAL samples and throughout 77 days in lung supernatants (Figure 7, solid line plots).

**Figure 8:**
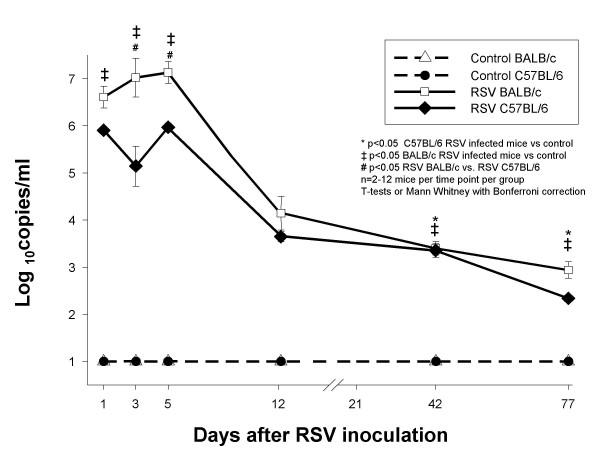
**Comparison of RSV loads measured by PCR in lung supernatants of BALB/c and C57BL/6 mice**. Groups of 2–12 BALB/c (△) and C57BL/6 (●) mice per group per time point were inoculated intranasally with sterile 10% EMEM (control), and were compared with RSV A2 infected Balb/c (□) and C57Bl/6 (◆) mice. Viral load was determined by PCR to detect RSV N gene. Data are presented as mean ± SEM Log10 PFU/ml of BAL. p < .05, by t-test when data normally distributed, or by Mann-Whitney Rank sum test when data were not normally distributed.

Additional experiments in both mouse strains demonstrated persistence of RSV RNA in lung supernatants for 77 days after inoculation (Figure 8) . Similar to the previous findings using plaque assays, RSV loads were greater in BALB/c than in C57BL/6 mice. Control mice of both strains had undetectable RSV load by RLT-PCR.

### 5. Correlations among disease severity markers, inflammatory indices, and viral load dynamics

Correlations were determined during both the acute phase of the disease, day 5, and during the progression to the chronic phase on days 14, 21 and 77 after inoculation.

During the acute phase in both mouse strains, airway obstruction (AO) peaked on day 5 and strongly correlated with histopathologic scores (HPS), BAL concentrations of RANTES, IFN-γ, MIP-1α and MIG, and RSV loads measured by both plaque assay in BAL samples and RLT-PCR in lung supernatants (Additional file 1). In BALB/c mice, AO also correlated with BAL TNF-α concentrations, RSV loads measured by plaque assay in BAL samples significantly correlated with those measured in lung supernatants by RLT-PCR in both C57BL/6 and BALB/c mice.

In addition, there were significant correlations between airway hyperresponsiveness (AHR) and HPS on day 14 in both BALB/c (r = 0.99, P = 0.0005, n = 4), and C57BL/6 mice (r = 0.85, P = 0.01, n = 7). AHR and HPS also correlated significantly on day 77 in C57BL/6 mice (r = 0.95, P = 0.012, n = 5).

In BALB/c mice alone, RSV loads measured by RLT-PCR correlated with AHR on day 14 (r = 0.69, P = 0.01, n = 12). RLT-PCR also correlated with AO on day 21 after RSV inoculation (r = 0. 71, P = 0.01, n = 12).

## Discussion

Intranasal inoculation of RSV induced acute and chronic effects on the respiratory dynamics and inflammatory response in C57BL/6 mice, similar to those previously reported in BALB/c mice [7]. The acute manifestations of RSV disease in C57BL/6 mice appeared similar but there were several differences compared with BALB/c mice. Acute airway obstruction was more severe and more prolonged in C57BL/6 than in BALB/c mice. BALB/c mice, however, demonstrated more severe histopathologic changes, greater viral loads, and BAL concentrations of most of the cytokines measured.

It is known that RSV infection causes variable degree of disease severity in different mouse strains [8] but there was no information on whether the different genetic backgrounds influence the long term outcome of RSV infection including chronic inflammation and persistent AO and AHR. The varied responses of these two mouse strains to different pathogens have been attributed in part to differences in their T helper (Th) lymphocyte response. Studies in mice infected with *Leishmania *showed that a Th2 skewed response caused more severe disease in BALB/c mice than in C57BL/6 mice that developed a Th1-dominant response [9]. In some localized bacterial infections, however, the severity of disease was milder in BALB/c mice and was described as Th1 skewed [10]. Indistinct Th responses were found in both mouse strains when infected with certain viruses [11]. Hence, the differences in the immune response might not only depend on the genetic background of the host, but also on the site and the specific pathogen involved in the infectious process. Several groups have demonstrated AHR in C57BL/6 mice during the acute stages of RSV infection. [22-24]. However, so far, our study is the first to shed light on the dynamics of inflammatory infiltrates, airway dysfunction (AO and AHR), and inflammatory cytokines during the long-term phase of RSV-induced disease, up to 77 days after primary infection in both C57BL/6 and BALB/c mice.

Researchers found that during RSV re-challenge following sensitization with different RSV proteins, mice developed a variable Th cytokine response [12-15] which was not exclusively dependent on the strain haplotype [16]. Our experiments, conducted in mice with primary RSV infection, demonstrated that IFN-γ, a Th1 cytokine, was elevated after RSV infection in both strains. IFN-γ was increased in the respiratory tract of children with acute RSV infection [17,18] and correlated with disease severity [19]. In mice, IFN-γ also correlates with the development of RSV disease [20]. Our studies demonstrate that RSV infection induces IFN-γ in both strains of mice and the magnitude of the response is not consistent with the traditional notion that BALB/c are Th-2 weighted and C57BL/6 are Th1-weighted. BALB/c mice showed greater IFN-γ response than C57BL/6 mice as previously shown by others [21]. In our experiments IFN-γ demonstrated the greatest correlation with disease severity (Additional file 1).

Like IFN-γ, the dynamics of TNF-α, RANTES, MIP-1α were comparable in both mouse strains. TNF-α has been linked to RSV disease severity [25]. In our model, BAL concentrations of TNF-α peaked shortly after RSV infection and correlated with markers of disease severity in BALB/c mice (Additional file 1), and were significantly greater in BALB/c than in C57BL/6 mice (Figure 5).

The CC chemokines, RANTES and MIP-1α, and the CXC chemokine MIG were also elevated in mice of both strains shortly after RSV inoculation. The biphasic production of RANTES, MIP-1α and MIG after RSV infection, with the second peak coinciding with the peak of viral replication and histological inflammation, has been previously described in BALB/c mice [7,26] but not in C57BL/6 mice. Although MIP-1α has been detected in C57BL/6 mice [27], no previous studies demonstrated the production of RANTES in the respiratory tract of these mice after RSV infection. These two chemokines correlate with acute disease severity both in humans and in mice. [19,28-30]. Increased production of MIG after RSV infection has been documented *in vitro *[31] and by us in BALB/c mice[7]. Others have linked MIG to persistent airway inflammation due to continuous chemotaxis of mononuclear cells [32]. In the present study we demonstrate significant correlation between BAL MIG concentrations and AO, and viral load dynamics in both strains of mice. Taken together, these results indicate that RSV induces severe acute pulmonary disease in both strains of mice despite different genetic backgrounds. Although there are quantitative differences during the acute phase of the disease, there were no significant distinctions in the pattern of cytokine responses, lung inflammation or clinical manifestations of disease, and both strains developed long-term airway disease defined by chronic inflammatory infiltrates and abnormal AHR. These findings provide further experimental support to the link between RSV and chronic airway disease in humans. We believe that this is the first description of RSV-induced long-term pulmonary disease in C57BL/6 mice.

The exact mechanism by which different inflammatory mediators participate in the pathogenesis of the acute RSV-induced disease process is not completely understood, and their potential role in determining the chronic consequences of RSV infection, such as AO, AHR and chronic inflammation is even less characterized. Studies in murine models and in humans have not demonstrated yet a direct relation between the acute inflammatory response to RSV and its chronic consequences in the respiratory tract pathology. Our studies demonstrated prolonged AO and persistent AHR in both mouse strains, long after any of the cytokines, potentially responsible for persistent inflammation that we measured, became undetectable in BAL samples. It is possible that the use of more sensitive methods, perhaps in specific compartments, will contribute to clarify the role of these molecules in the chronic respiratory function abnormalities induced by RSV.

Until recently, it was suggested that initial RSV infection, although of short duration, was sufficient to elicit an exaggerated inflammatory response which would evolve into chronic inflammation and long-term airway abnormalities. An alternative, but not contradictory explanation for the development of chronic airway disease could be related to long-term antigenic stimulation. In this hypothesis, the presence of low level viral infection could represent a persistent stimulus responsible for the chronic inflammatory changes and the lasting pulmonary function abnormalities. The application of the RLT-PCR assay to this model demonstrated the persistence of RSV RNA in the respiratory tract for 11 weeks after inoculation in both strains of mice and was also associated with long-term pulmonary disease.

RSV can persist and replicate *in vitro *[33-35]. The persistence of RSV genomic RNA sequences and viral antigens has been described in guinea pigs for several weeks after acute primary infection [36-38]. Bovine RSV infection in calves resulted in persistent detection of RSV N gene for several weeks [39]. Miller et al. reported detection of G protein RNA by RLT-PCR in lungs of BALB/c mice up to day 12 after inoculation. [26].

It appears unlikely for a RNA virus to persist without at least low-level replication in the host. This is supported by the studies by Schwarze et al., who found RSV genomic and messenger RNA in mouse lung homogenates for 100 days after inoculation, and were able to recover low titer virus in culture after treatment with anti-CD4 and anti-CD8 antibodies [40].

Clinical studies have demonstrated that adults with stable COPD without acute exacerbation had RSV RNA detected by PCR in the upper respiratory tract [41]. The persistence of RSV RNA in children has not been extensively studied. Post-mortem specimens of children who died of sudden infant death syndrome showed positive results by PCR targeting the RSV N gene in 27% of cases and in 18% of the controls. Since some children died during summer the authors suggested the possibility of viral persistence [42]. The persistence of RSV in the respiratory tract of children and its potential contribution to the development and maintenance of AHR is a critical question that needs to be addressed.

In summary, we provide evidence that RSV-induced acute and chronic airway disease is reproducible in two genetically distinct mouse strains. The long-term airway disease coincided with persistent detection of RSV genome in the respiratory tract. It is yet unclear whether the persistence of RSV RNA plays a role in the development of chronic airway disease. Further studies are needed to characterize the potential mechanisms that would allow viral persistence and its possible contribution to the pathogenesis of RSV-induced long-term pulmonary disease.

## Materials and methods

### Mice

Female, 7–8-week old pathogen-free C57BL/6 and BALB/c mice (Charles River Laboratories, Wilmington, MA) were maintained in filter top cages, and routinely monitored for other pathogens [7]. After inoculation, all mice were kept under the same conditions and were provided identical care. Sentinel mice housed in the mouse storage room are routinely used for health surveillance. Sentinel mice had no detectable antibodies against mouse hepatitis virus, Sendai virus, pneumonia virus of mice, reo-3 virus, mouse encephalitis virus (GD-7), mouse rotavirus (EDIM), minute virus of mice, and *Mycoplasma pulmonis; *screening for pinworm and mites was also negative. This study was approved by the Institutional Animal Care and Use Committee at the University of Texas Southwestern Medical Center at Dallas.

### Virus

RSV A-2 strain was maintained in our laboratory as described [7]. Results of plaque assays were reported in Log_10 _PFU/mL, with 1.7 Log_10 _PFU/mL being the lowest limit of detection.

### Inoculation

Mice were anesthetized using inhaled methoxyfluorane and intranasally inoculated with 10^7 ^PFU of RSV in 100 μl of 10% Eagle's minimal essential medium (EMEM) [6]. Uninfected control animals were sham-inoculated with 100 μl of sterile 10% EMEM. Animals were allowed 30 seconds to aspirate the inoculum while held upright until fully recovered from the anesthesia.

### Plethysmography

Unrestrained, whole-body plethysmography (Buxco Electronics, Inc. Sharon, CT) was used to measure the Enhanced Pause (Penh) to evaluate airway obstruction (AO) and airway hyperresponsiveness (AHR), as previously described [7,43]. Briefly, mice were allowed to acclimate to the chamber, and then plethysmograph readings were recorded to establish baseline Penh values to determine AO. Next the mice were exposed to aerosolized methacholine (Sigma; 50 mg/ml) for 4 minutes; after exposure, plethysmograph readings were recorded again to determine AHR. Groups of infected and control mice were always evaluated in parallel at all time points during the entire study. Preliminary studies showed that normal C57BL/6 mice have greater baseline Penh values than normal BALB/c mice; therefore, groups of infected and control mice of each strain were evaluated in parallel during the entire study. Plethysmography was performed in the groups of mice to be sacrificed for sample collection at each time point, and in an additional group of mice that were followed throughout each experiment. A total of 4 independent experiments including 10–30 mice per group per time point were conducted.

### Sample collection

Mice were anesthetized with an intraperitoneal injection of 75 mg/kg of ketamine and 5 mg/kg of acepromazine before euthanasia by exsanguination. On average, 4–12 mice were sacrificed per group per time point. Bronchoalveolar lavage (BAL) specimens were obtained as described previously [7,43] to measure cytokine concentrations and RSV loads. Histologic evaluation was performed in whole-lung specimens fixed with a 10% buffered formalin solution. Whole lung samples were also collected from another group of mice for determination of viral loads by plaque assay and by real-time polymerase chain reaction (RLT-PCR). Specimens were obtained on days 0 (within 2 hours of inoculation), 1, 3, 5, 8, 9, 12, 14, 21, 28, 42, and 77 post-inoculation.

### Histopathology

Formalin fixed lungs were paraffin embedded, sectioned and stained with hematoxylin and eosin. Histopathologic score (HPS) was determined by a pathologist, unaware of the infection status of the animals. Each section was graded on the basis of a cumulative score from 5 categories: (1) peribronchiolar and bronchial infiltrates, (2) bronchiolar and bronchial luminal exudates, (3) perivascular infiltrates, (4) the number of monocytes, and (5) parenchymal pneumonia. This HPS system assigns values from 0 to 21. This scoring system has been previously validated in RSV infection [7,43].

### BAL cytokines

Concentrations of TNF-α, IFN-γ, IL-4, IL-10, MIG, RANTES, and MIP-1α were measured in BAL specimens by ELISA (R&D Systems, Minneapolis, Minn.) for up to 14 days after inoculation. The lower limit of detection for each of these assays were: 120 pg/mL for TNF-α, 50 pg/mL for IFN-γ, 40 pg/mL for IL4, 16 pg/mL for IL-10, 16 pg/ mL for MIG, 200 pg/mL for RANTES and 60 pg/mL for MIP-1α. For statistical analysis, samples with optical density readings below the limit of the standard curve of the assay were assigned a value half that of the detection level.

### RSV loads by plaque assay and Real-time Polymerase Chain Reaction (RLT-PCR)

Plaque assay tissue culture was used to measure viral loads in BAL specimens and lung homogenate supernatants as previously described [7]. Lung supernatant samples were prepared by placing whole lung specimens in 1 mL of 10% EMEM, on ice. Lungs were homogenized using sterile probes and a rotor tissue homogenizer (Omni International Inc., Marietta, GA). Lung supernatants were separated by centrifugation at 2000 g for 10 minutes at 4°C and 100 μL were used immediately for determination of viral loads by plaque assay; the remaining supernatant was frozen at -80°C for viral load measurements by RLT-PCR. In a subset of experiments, lung samples used for RLT-PCR were placed in 1.5 mL of RNA stabilization reagent (RNA later, Qiagen Inc., Valencia, CA) immediately after sample collection. Lung supernatants were then obtained by homogenization and centrifugation. The RLT-PCR viral load values measured using this method were similar to those obtained when no RNA later was used; therefore, all results of lung homogenate supernatants are presented together. One sample per mouse was evaluated as a single specimen. Quantitative RLT-PCR was used targeting the conserved region of the RSV N-gene. Forward (5'-AGA TCA ACT TCT GTC ATC CAG CAA) and reverse (5'-TTC TGC ACA TCA TAA TTA GGA GTA TCA AT) primers amplified an 85-bp region containing the 25-mer FAM-labeled probe (5'-CAC CAT CCA ACG GAG CAC AGG AGA T), as previously described [44]. RSV RNA was extracted from 1 mL of lung supernatant samples using ion-exchange mini-columns (Qiagen RNeasy Mini Kit, Valencia, CA, USA) and cDNA was prepared by reverse transcription using 2.5 uM random hexamers for 10 min at 22°C, 30 min at 42°C and 5 min at 95°C. Real-time PCR was performed using a Perkin-Elmer /Applied Biosystems 7700 sequence detector (Foster City, CA) using 10 μl cDNA/ in a total volume of 50 μl master mix with the following run conditions: 1 cycle for 2 min at 50°C and 10 min at 95°C each, followed by 50 cycles for 15 seconds at 95°C and 60 seconds at 60°C. RSV A known concentrations were used to derive a standard curve. Standards and negative controls were run together with each PCR assay. The lower limit of detection of the assay was 10 viral copies/mL.

### Statistical methods

T-test or Mann-Whitney Rank Sum test were used according to data distribution for comparisons between groups and the Spearman Rank Order test was used for correlations, as all the data taken together were not normally distributed, using the SigmaStat^® ^(SPSS, Inc. Chicago, Illinois) software package.

## Abreviations used

PCR polymerase chain reaction

TNF tumor necrosis factor

IL interleukin

IFN interferon

MIP macrophage inflammatory protein

RANTES regulated on activation, normal T-cell expressed and secreted

MIG monokine induced by interferon-gamma

## Competing interests

The author(s) declare that they have no competing interests.

## Authors' contributions

SCB study design, RSV infection, data analyses; AM BAL, formalin fixation, plethysmography; AMG histopathology and special staining; KDO real-time PCR; AMR and MFA plethysmography and BAL; OR data interpretation; HSJ project design, experimental analyses and interpretation.

## Supplementary Material

Additional File 1 **Significant Correlations on Day 5 after RSV Inoculation in C57BL/6 and BALB/c mice**. r = correlation coefficient; p = p-value, n = number of samples analyzed. Spearman's rank order analysis was used. Statistically significant correlations (p < 0.05) listed in bold font. N/A, not applicable, samples from different animals; * TNF-α concentrations undetectable on day 5. Click here for file
